# Supplementary figures and images for: Discovery of diverse Pectocaris species at the Cambrian series 2 Hongjingshao formation Xiazhuang section (Kunming, SW China) and its ecological, taphonomic, and biostratigraphic implications
Source: PeerJ. 2024 Apr 15;12:e17230. doi: 10.7717/peerj.17230 (PMC11025544; doi:10.7717/peerj.17230)

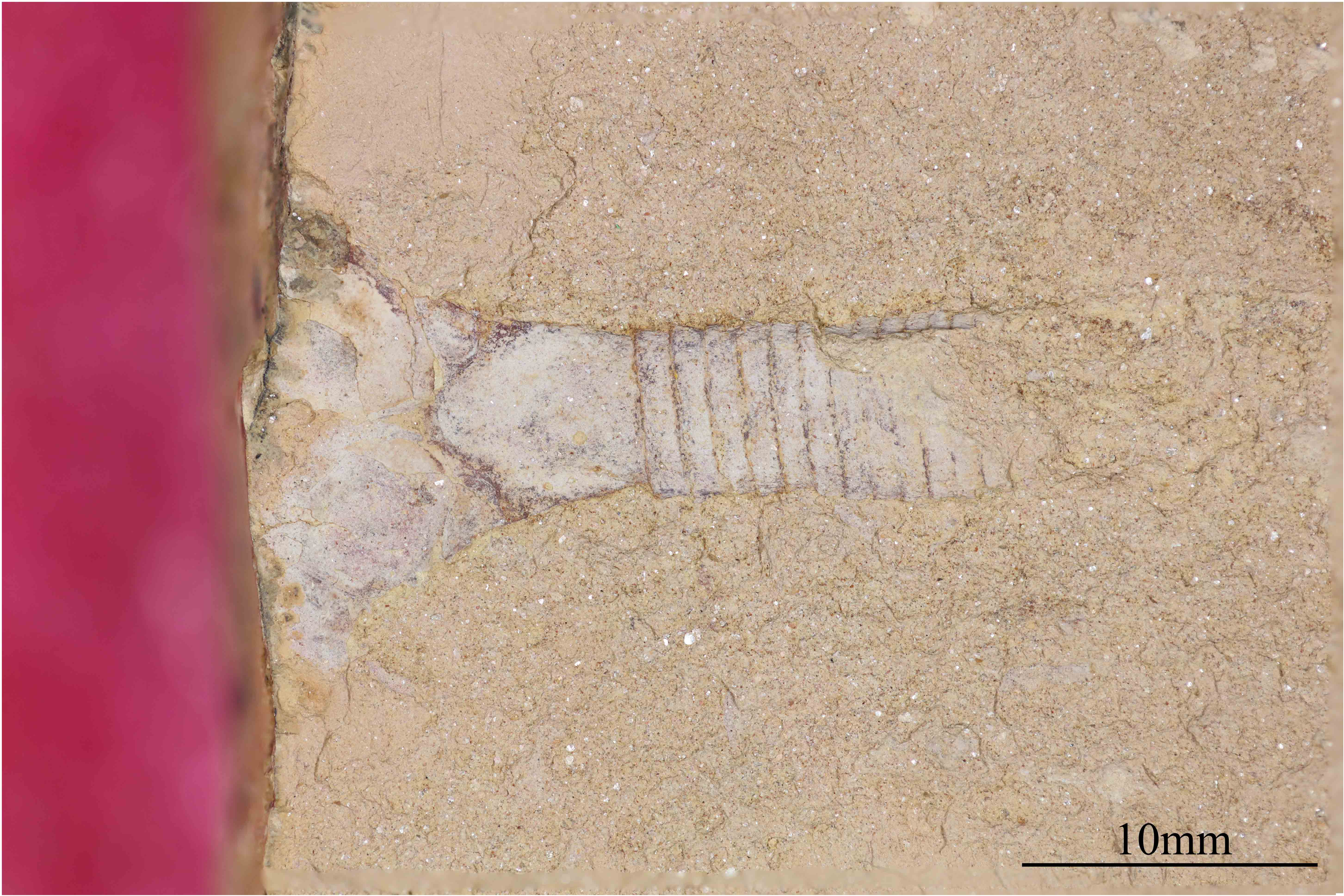

Supplement: Supplemental Information 1 [file peerj-12-17230-s001.jpg]

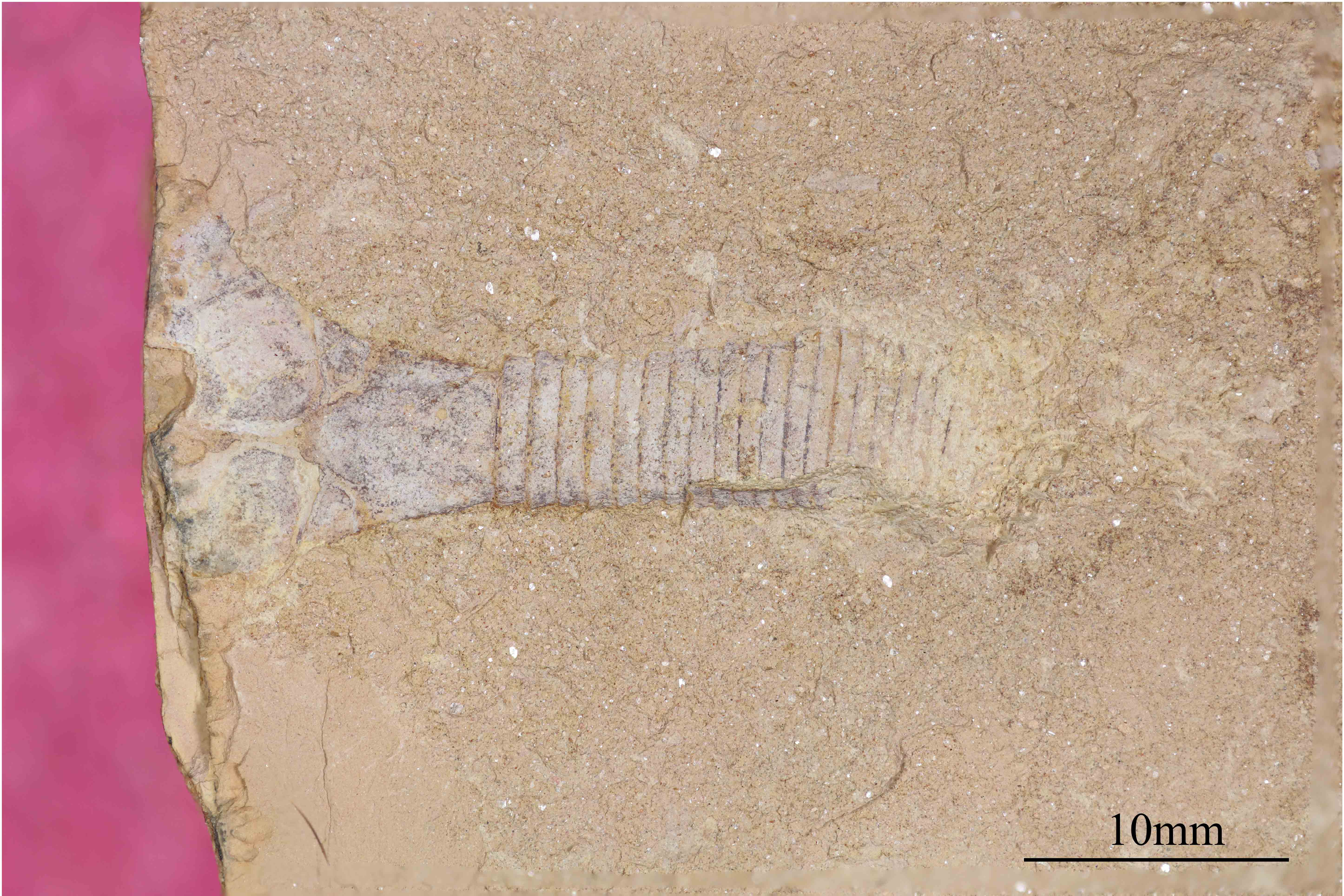

Supplement: Supplemental Information 2 [file peerj-12-17230-s002.jpg]

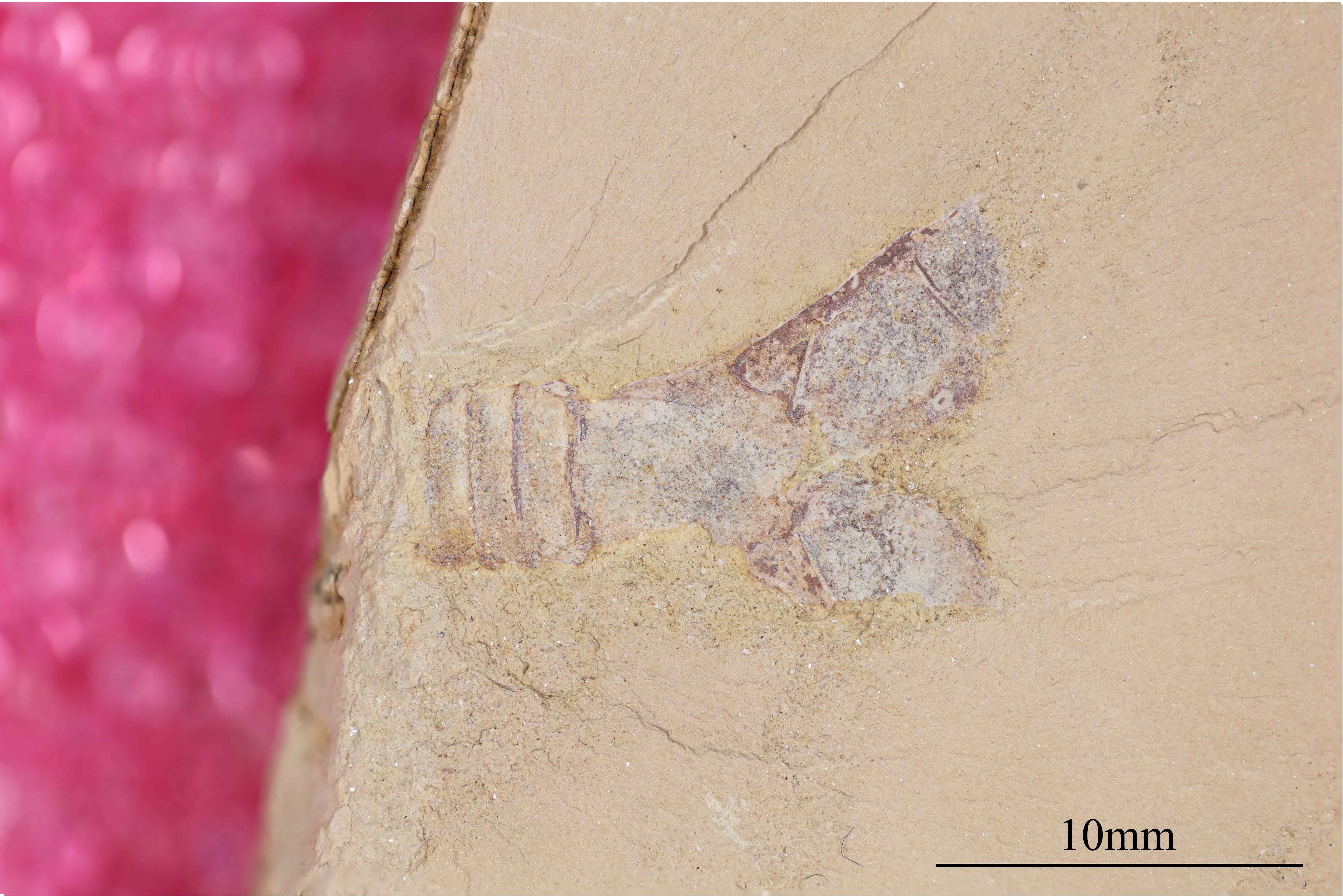

Supplement: Supplemental Information 3 [file peerj-12-17230-s003.jpg]

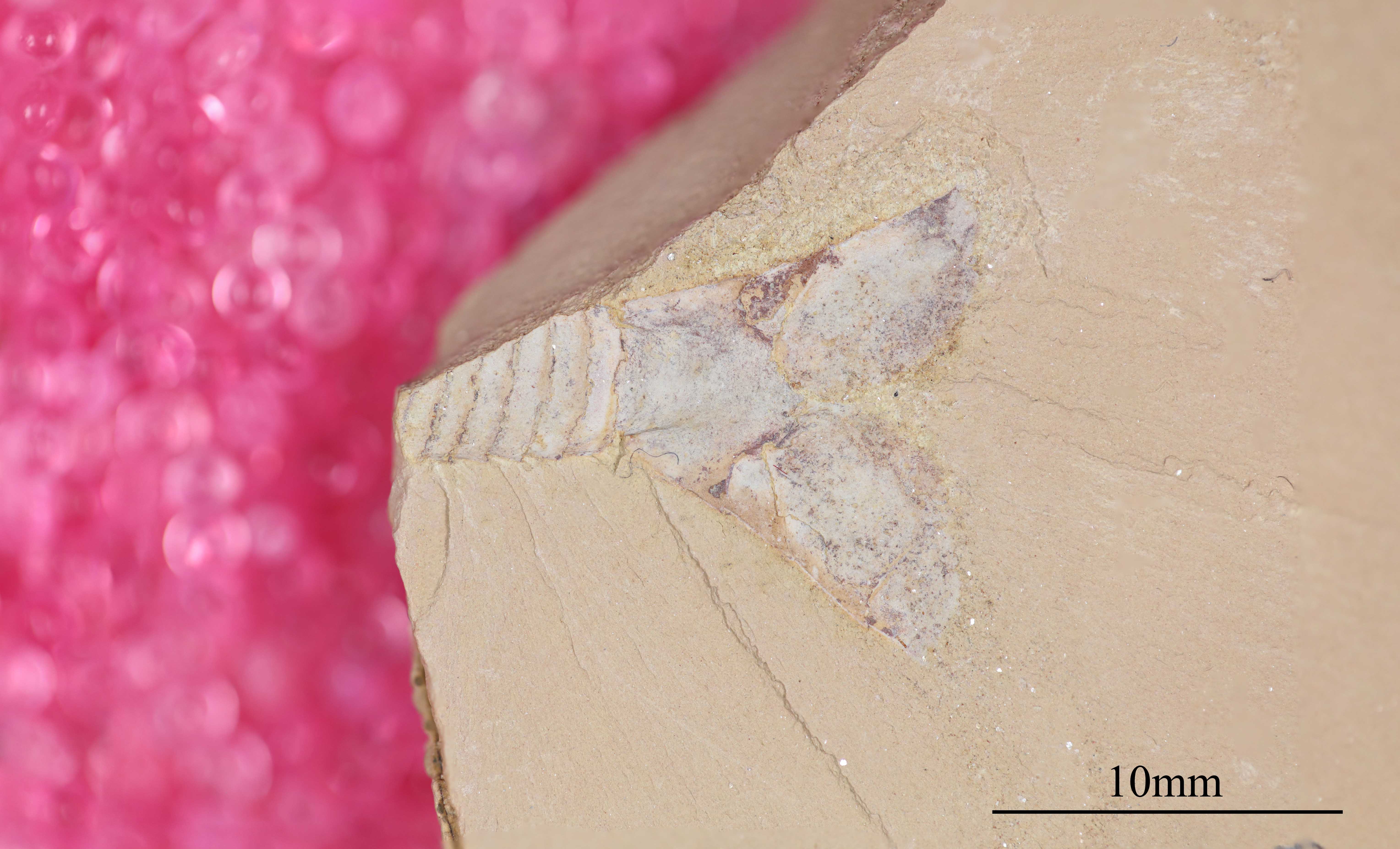

Supplement: Supplemental Information 4 [file peerj-12-17230-s004.jpg]

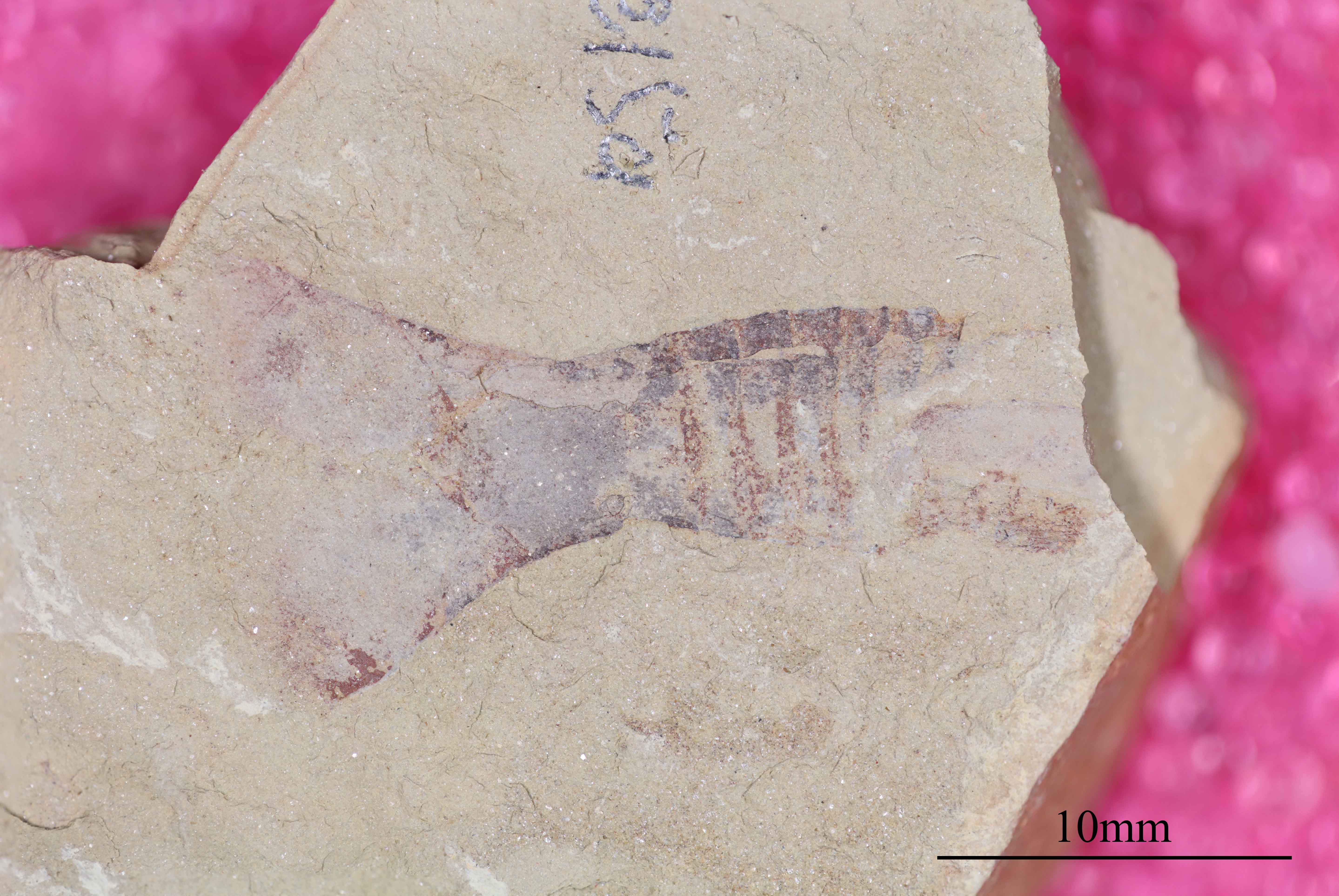

Supplement: Supplemental Information 5 [file peerj-12-17230-s005.jpg]

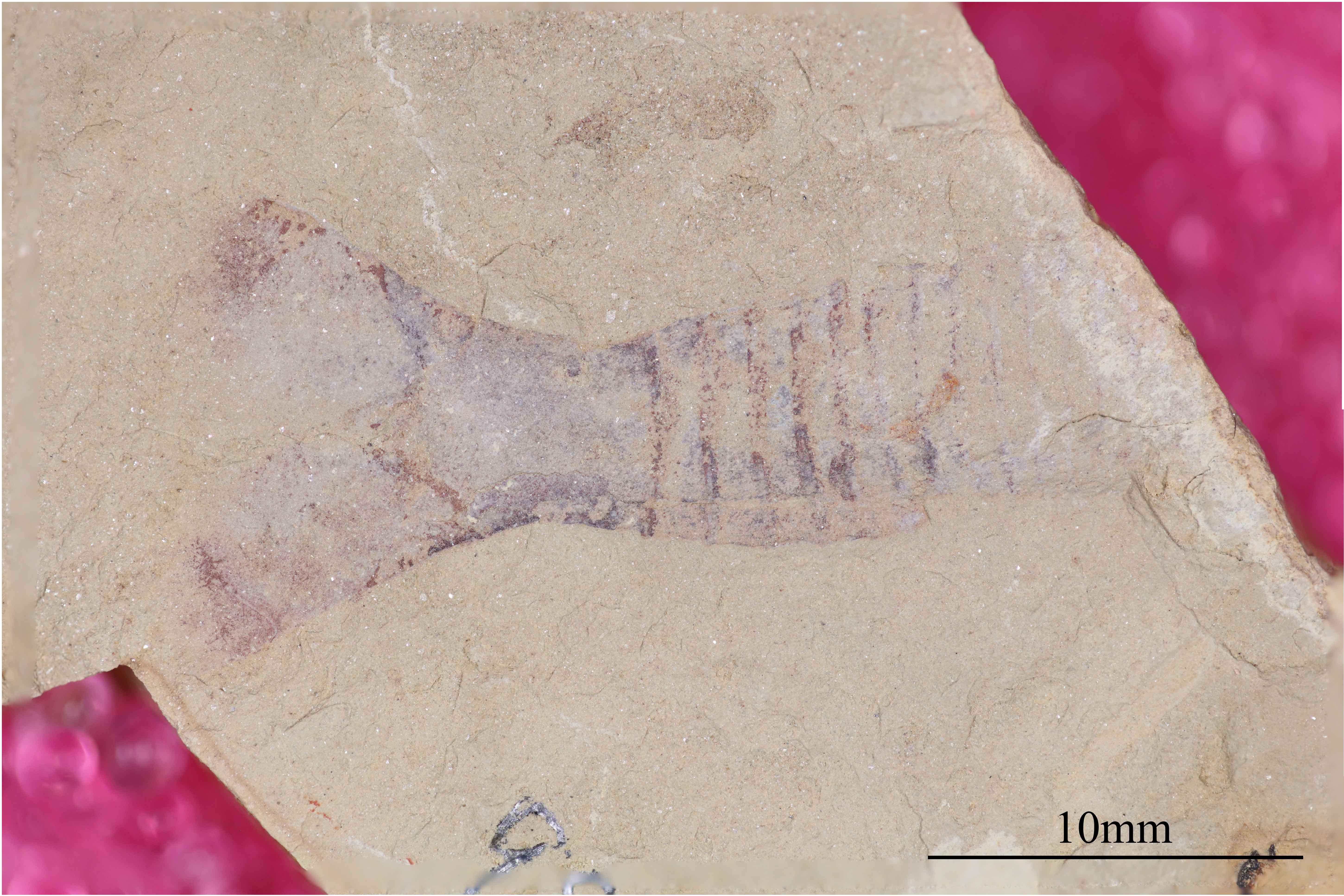

Supplement: Supplemental Information 6 [file peerj-12-17230-s006.jpg]

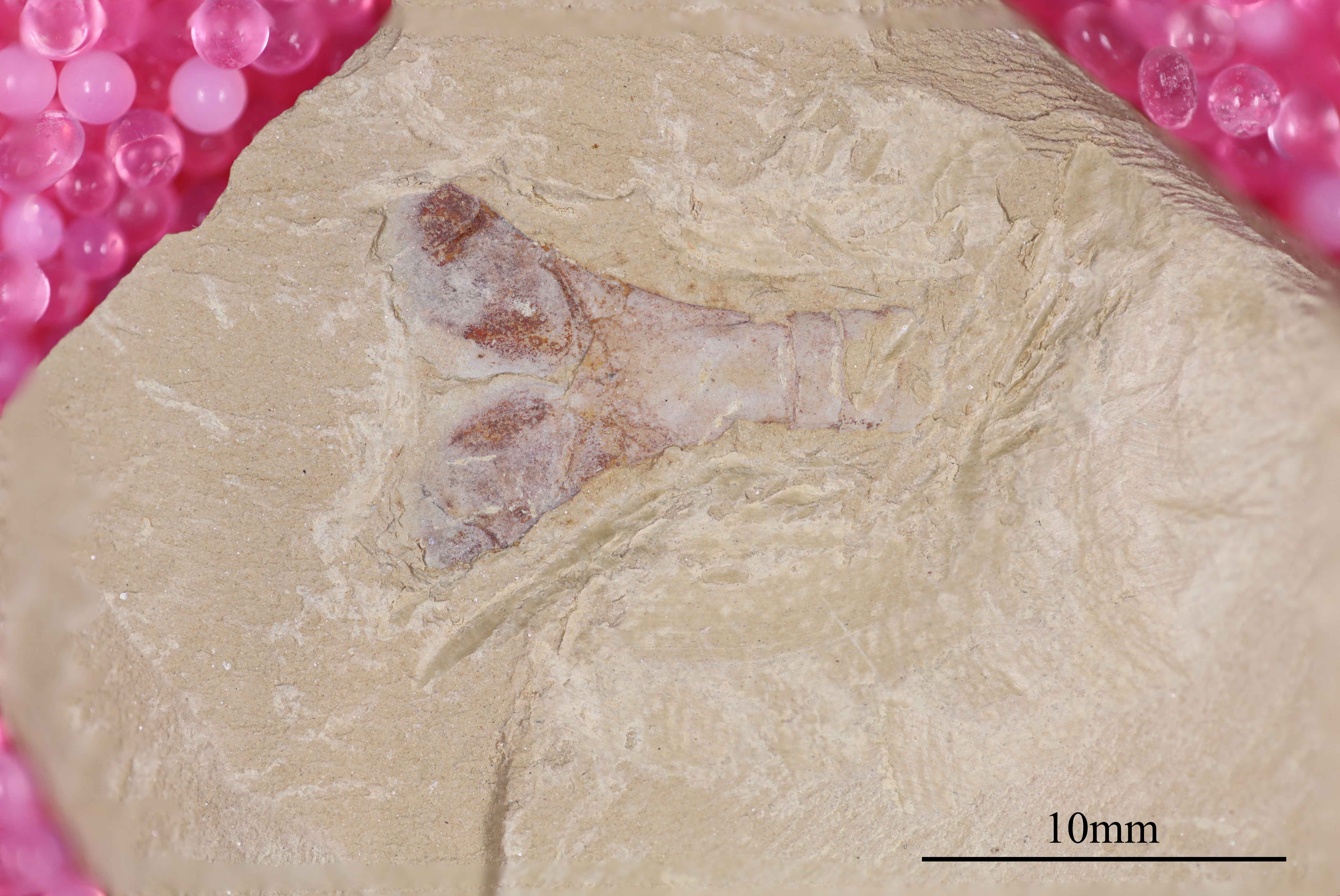

Supplement: Supplemental Information 7 [file peerj-12-17230-s007.jpg]

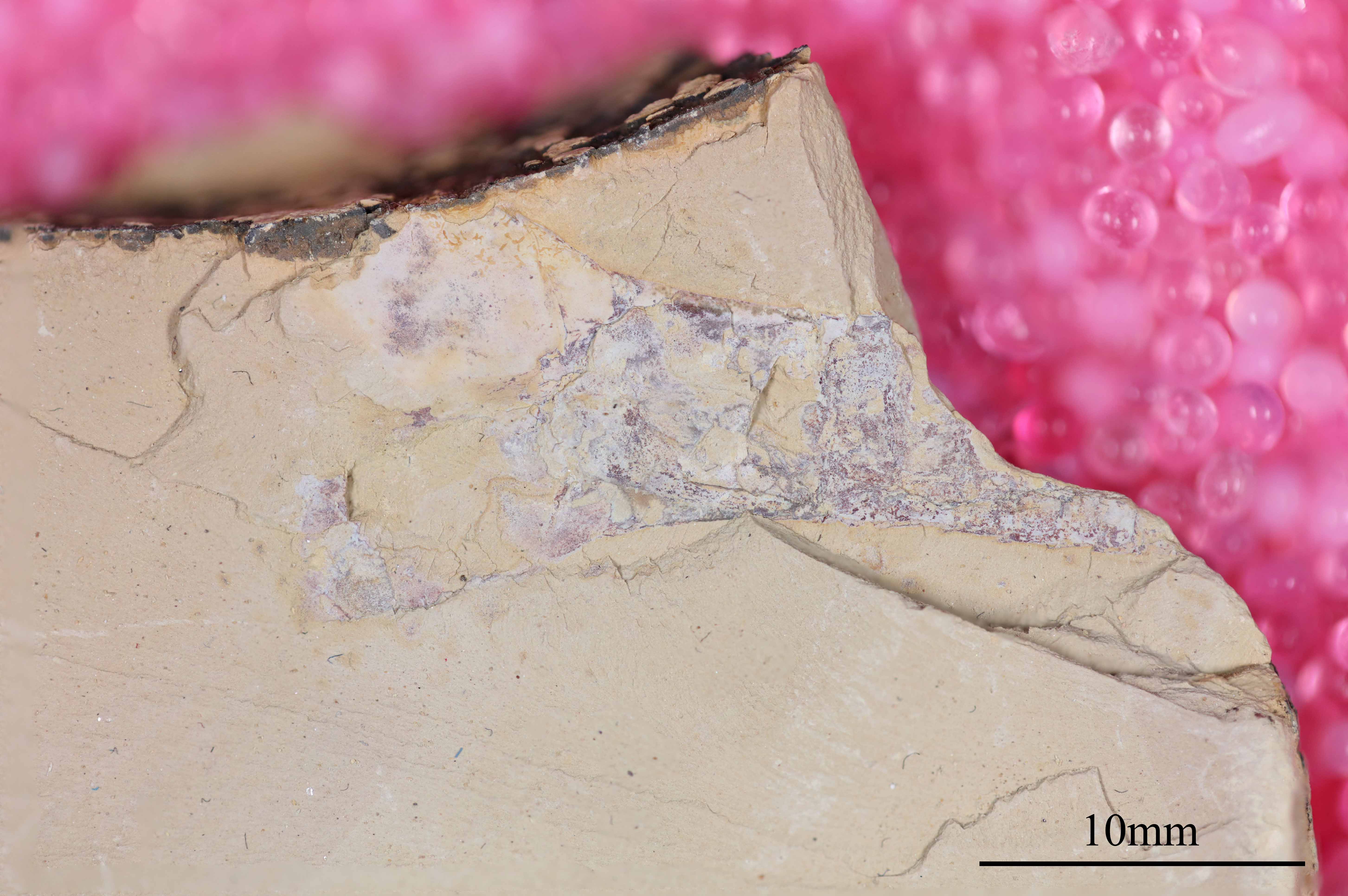

Supplement: Supplemental Information 8 [file peerj-12-17230-s008.jpg]

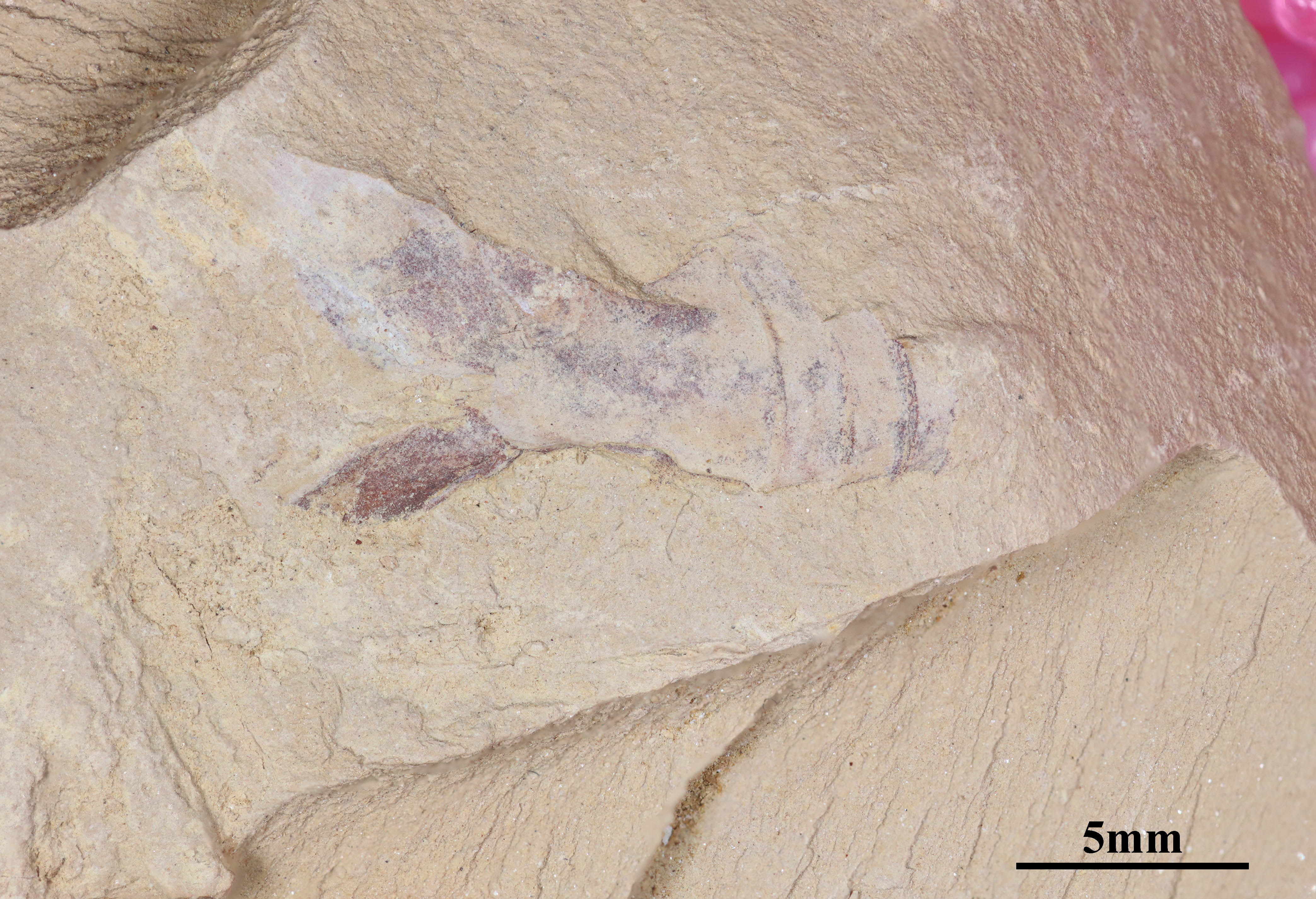

Supplement: Supplemental Information 9 [file peerj-12-17230-s009.jpg]

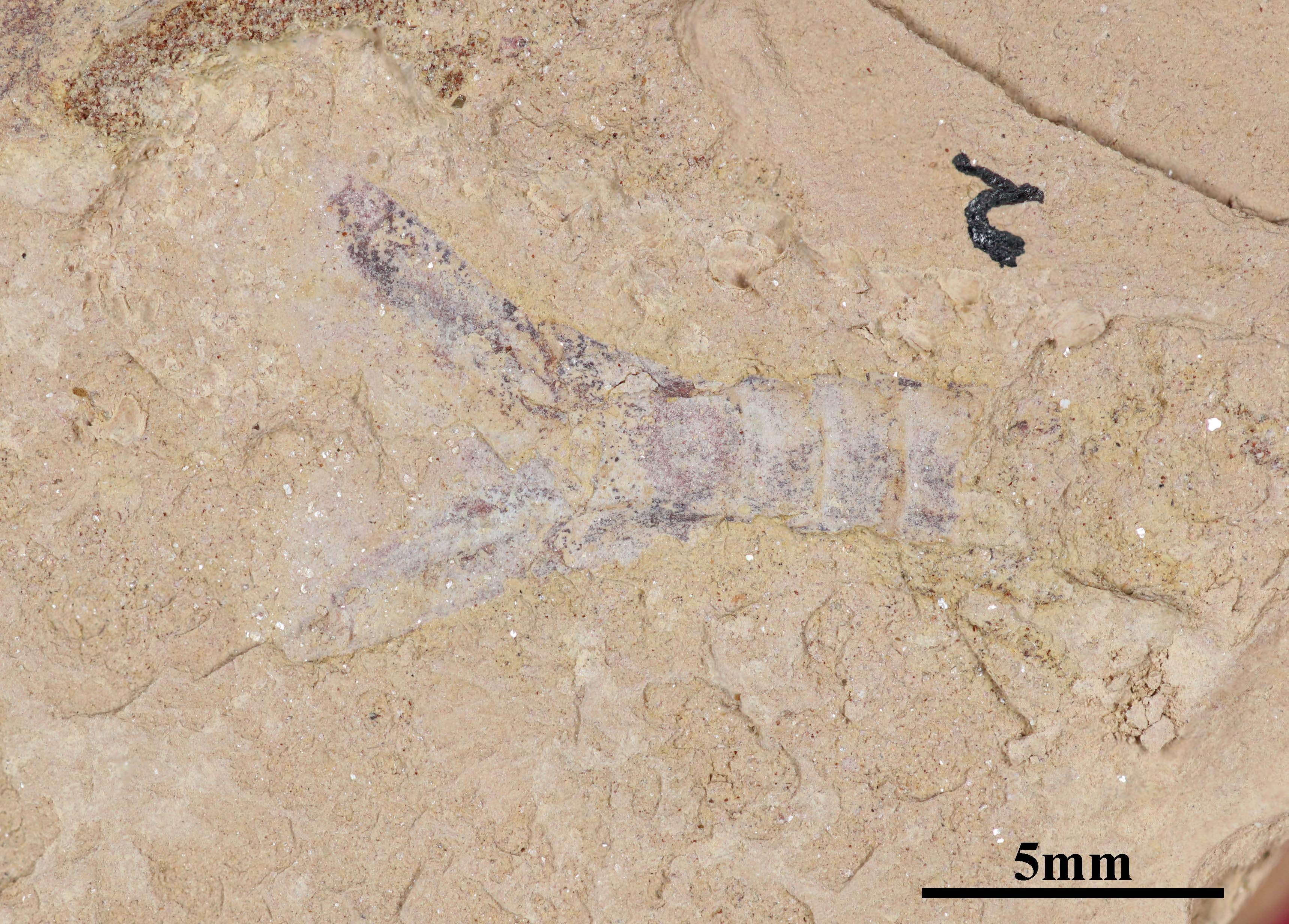

Supplement: Supplemental Information 10 [file peerj-12-17230-s010.jpg]

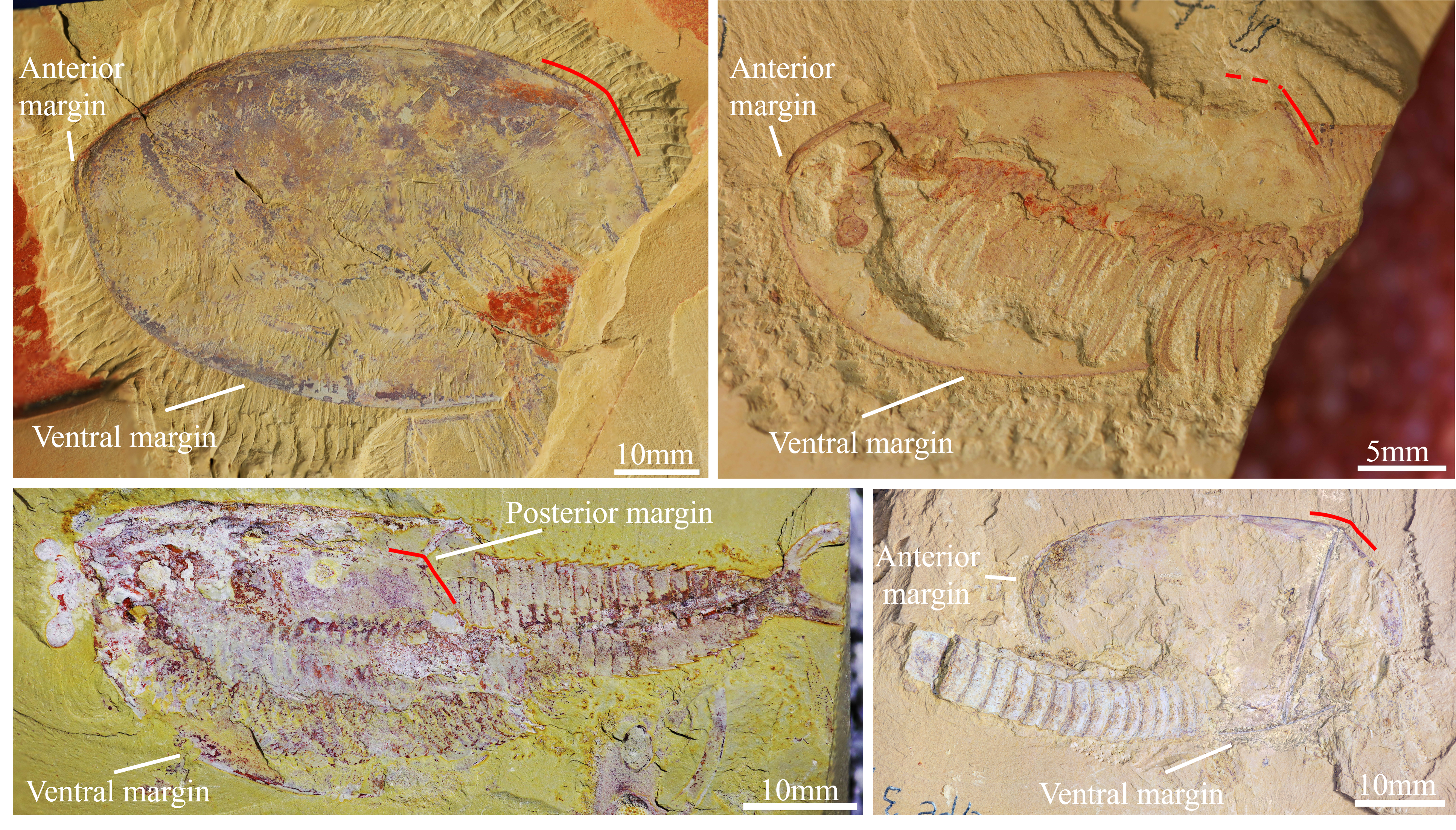

Supplement: Supplemental Information 11 [file peerj-12-17230-s011.png]
